# Evolution of oxygen utilization in multicellular organisms and implications for cell signalling in tissue engineering

**DOI:** 10.1177/2041731411432365

**Published:** 2011-11-16

**Authors:** Katerina Stamati, Vivek Mudera, Umber Cheema

**Affiliations:** 1 Stanmore Campus, UCL, London, UK; 2 Institute of Orthopaedics and Musculoskeletal Sciences, UCL Division of Surgery & Interventional Science, University College London, Stanmore Campus, UK

**Keywords:** angiogenesis, evolution, oxygen, physiological hypoxia, stem cells

## Abstract

Oxygen is one of the critically defining elements resulting in the existence of eukaryotic life on this planet. The rise and fall of this element can be tracked through time and corresponds with the evolution of diverse life forms, development of efficient energy production (oxidative phosphorylation) in single cell organisms, the evolution of multicellular organisms and the regulation of complex cell phenotypes. By understanding these events, we can plot the effect of oxygen on evolution and its direct influence on different forms of life today, from the whole organism to specific cells within multicellular organisms. In the emerging field of tissue engineering, understanding the role of different levels of oxygen for normal cell function as well as control of complex signalling cascades is paramount to effectively build 3D tissues *in vitro* and their subsequent survival when implanted.

## Discovery of oxygen

In the 1600s, scientists began to realize that there was an element in the air that was essential for life. This element could be effectively depleted in an enclosed chamber in the presence of a naked flame, and it could have severe consequences for a mouse within the same chamber.^1,2^ The scientist, John Mayow, proved that a component part of air was critical for life and could be removed by a naked flame and respiration. Work a century later showed that mice were able to survive for longer in air heated by mercury oxide, compared to air heated using a flame, again pertaining to the fact that fire required an element in air, and by utilizing this element, its depletion directly resulted in the demise of a mouse. This work was repeated and the results essentially described a component termed ‘eminently breathable air’, later termed ‘oxygen’.^1,2^

## Changes in atmospheric oxygen

**Figure 1.**
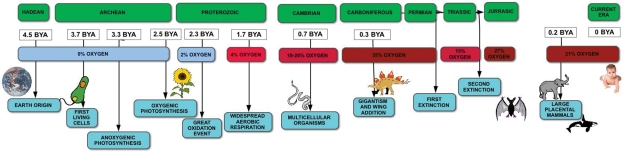
Oxygen change and the evolution of life on Earth. Oxygen concentrations corresponding to different eras are indicated with corresponding events in life evolution. BYA: billion years ago.

Air contains a mixture of gases, which include approximately 78% nitrogen, 21% oxygen and 0.03% carbon dioxide (at sea level). Fluctuations in these levels have occurred multiple times throughout the evolution of earth’s atmosphere (Figure 1). It is known that Earth was once an anoxic environment but there was a transition to an oxic atmosphere around 2.3 billion years ago (Figure 1).^3–5^

Complex life on Earth began approximately 0.5 billion years ago, although geologically the planet was formed 4.5 billion years ago. The first organisms (mainly bacteria) appeared 3.7 billion years ago (Figure 1). These organisms had to survive in anoxic conditions and therefore used anoxygenic photosynthesis for energy, using hydrogen or sulphur for this process, rather than water.^2^

During the Hadean eon (4.6–3.8 billion years ago), when life was non-existent, oxygen levels were nearly zero. As cyanobacteria are the earliest source of oxygen production, it was generally thought that the emergence of this life form predated any form of aerobic respiration. Evidence now suggests that there was limited aerobic respiration prior to the emergence and evolution of cyanobacteria.^3,6,7^ This was possible via the disproportion of atmospherically derived H_2_O_2_ by early catalase enzymes (2H_2_O_2_ → 2H_2_O + O_2_) to prevent the formation of their harmful reaction products, which include hydroxyl radicals.^6,8^ This form of aerobic respiration was, however, disproportionately low compared to the predominant anaerobic respiration.

The ‘great oxidation event’, which occurred in the early Proterozoic period (2500–2542 million years), caused the rise of oxygen to a maximum of 2% pO_2_. During the Cambrian (542–488 million years), the presence of plants is believed to have influenced oxygen levels increasing them substantially, possibly as high as 10%–20%.^1^

In the late Paleozoic era (542–251 million years), oxygen is believed to have reached 35%. This oxygen rise occurred whilst nitrogen remained constant, increasing the total atmospheric pressure. It is assumed that 35% is the highest value possible, since combustion of the biosphere would occur beyond such oxygen and nitrogen levels.^1^ This was followed by fluctuations in oxygen levels from 15% to 27%.^1,9,10^ The present 21% oxygen has remained stable for the last 350 million years.^1^

Photosynthesis is the dominant mechanism for replenishing oxygen. Photosynthesis evolved in the precursors of cyanobacteria, producing oxygen that accumulated in the atmosphere at the time.^3,4^ All photosynthesizing organisms have been found to contain the same inorganic core that allows the splitting of water into oxygen, protons, and electrons.^2,4^ These organisms reduce water as in the reaction H_2_O → 2H^+^ + 2e^−^ + 1/2 O_2_.^2^ During oxygen production by oxygenic photosynthesis, ~0.1% of the oxygen produced is supplied into the atmosphere. It has been calculated that 1 mol of oxygen remains in the atmosphere or in the ocean for approximately 3.25 million years.^1^

Oxygen reservoirs can be found in the atmosphere, the ocean surface and the deep ocean.^11^ Although oxygen begun accumulating in the atmosphere during the Proterozoic, it is estimated that low levels of oxygen were already present in the surface of ocean waters, produced through water photolysis.^3^ Deep ocean oxygenation is dated approximately 1800 million years ago^12^ with the timing of these events based on the study of carbonate precipitates within oceans. The absence of oxygen in sea water allowed high levels of Fe^2+^, whereas Fe^2+^ levels decreased during oxygen-rich eras. The difference in iron levels affected the texture of the precipitates that formed within oceans, with micrite precipitation during anoxic eras and calcite precipitation during high-oxygen–low-iron eras, allowing scientists to deduce oxygen levels during different eras.^13^

## From prokaryotes to eukaryotes

The appearance of eukaryotic cells ~2 billion years ago has been linked to the introduction of oxygen in the atmosphere and therefore aerobic metabolism.^4,14^ The increased presence of oxygen produces a more efficient energy source in the form of aerobic metabolism, producing 16–18 times more adenosine triphosphate (ATP) per hexose sugar than anaerobic metabolism.^4,15^ Since aerobic metabolism generates more energy, approximately 1000 more reactions can occur than under anaerobic metabolism.^16^ This allowed the generation of new metabolites, for example, steroids, alkaloids and isoflavonoids.^15^ Steroids and polyunsaturated fatty acids are important elements of membranes; thus, they must have been involved in promoting organelle formation and cell compartmentalisation.^17^

As some of the metabolites produced from respiration are involved in processes that target nuclear receptors, it has been hypothesized that higher ambient oxygen promoted these nuclear signalling systems within cells. Nuclear factors have conserved volumes and are highly hydrophobic, since they must pass through cell membranes. Experiments comparing the volume and hydrophobicity of both aerobic and anaerobic metabolites to those known for nuclear ligands indicate that aerobic metabolites are more hydrophobic and more closely match the required volumes of appropriate molecules for nuclear factors compared to the anaerobic molecules.^15^ Since these appear important in superior eukaryotes, it has been hypothesized that such events influenced by increased oxygen levels have influenced biological evolution.

Eukaryotes assign more communication roles to proteins than prokaryotes, allowing for more complex and variable signalling pathways. In order to achieve this, various changes in protein structure and function had to occur during evolution. The secondary structures of transmembrane proteins are more hydrophobic, and oxygen as well as nitrogen is important during the formation of hydrophilic structures. Acquisti et al.^14^ studied the oxygen content and the topology of transmembrane proteins of different organisms. The ratio of receptors to channels was higher, with a greater amount of oxygen-rich proteins, in more highly developed and ‘recent’ organisms. For example, approximately 68% of transmembrane proteins in humans are high-oxygen content, whereas in most bacteria, such as *E. coli*, only 36% are oxygen rich. The size of the extracellular domains of transmembrane proteins increased as ambient oxygen concentration increased.^14^ Larger and higher oxygen-containing proteins are more energy demanding than those lacking oxygen in their side chains. However, the primary hypothesis suggests that the presence of large and oxygen-rich amino acids as side chains would have resulted in weak protein structures during anoxic eras.^14,18^

Eukaryotes have an abundance of oxygen in the plasma membrane, as oxygen is utilized in the mitochondria. This compartmentalisation may have evolved as a mechanism to protect the transmembrane proteins, which are rich in oxygen. Through the development of complex compartmentalisation of cells, multiple processes including signalling and oxygen levels could also be controlled in different parts of the cell. In addition, there has been the emergence of multiple cell types within the same ‘greater’ organism, with over 200 different cell types in the adult human body. Multicellular organisms may have required both the accumulation of oxygen-rich amino acids in their transmembrane proteins and the allocation of respiration to specific intracellular compartments, that is, mitochondria.^18^

## Oxygen toxicity

Although oxygen is essential for life in most organisms, some of its metabolic products can be toxic. Dioxygen (O_2_) is ‘paramagnetic’ and has two electrons with ‘parallel spins’.^1^ This renders reactions with O_2_ difficult, since the organic donor has to undergo a ‘slow spin inversion’ so that it can donate its electrons. This is not the case with singlet oxygen. In order to circumvent this necessity, O_2_ is either combined with a paramagnetic metal, for example, Cu or Fe, or electrons are added to oxygen in stages. This means that O_2_ is reduced to H_2_O by producing the superoxide anion radical (^⋅^O_2_^−^), hydrogen peroxide (H_2_O_2_) and hydroxyl radical (^⋅^OH). These intermediates, called free radicals, can pose a threat to cells. Hydrogen peroxide can be produced by superoxide, OH can interact easily with organic compounds and singlet oxygen reacts with carbon–carbon double bonds damaging cell membranes through a free radical reaction with fatty acids.^1^

Since these products are toxic to cells, enzymes that contain paramagnetic transition metals help avoid the production of these intermediates. For example, cytochrome c oxidase involved in the reduction of oxygen to water has iron and copper at its active sites. In addition, there are other enzymes that are responsible for catalysing the reactions that involve hydrogen peroxide and hydroxyl radicals, in order to minimize their effects. For example, catalases and glutathione peroxidases catalyse the reduction of hydrogen peroxide to water. Superoxide dismutases are the catalysts when O_2_^−^ is transformed to H_2_O_2_.^1^ These enzymes provide the first mechanism of defence in cells, and other antioxidants are responsible for minimizing any further damage. For example, vitamins A, C and E act as such compounds and stop the free radical chain reactions that damage cell membranes.^1^

The defence mechanisms against these toxic elements are thought to have evolved before the rise in oxygen levels that allowed for aerobic respiration. If these mechanisms had not been in place, there could have been deleterious effects on the first oxygen-respiring organisms. For example, cytochrome oxidase, the catalyst in oxygen to water reduction, was found before oxidative phosphorylation began. In addition, carotenoids, which do not require oxygen for their production and are produced by anaerobes, protect against the effects of H_2_O_2_. What led to the appearance of these elements prior to the rise in oxygen is unknown, but a source other than oxygen itself must have driven the process. Once oxygen levels rose, further defence mechanisms evolved.^1,6^

## Organism changes throughout evolution as a result of oxygen fluctuations

There is an association between evolution and changes in atmospheric oxygen concentration. Although not a sole contributor to these changes, endothermy, placentation and animal size were all affected by oxygen increases.^19^

During the Carboniferous era (359–299 million years), terrestrial invertebrates (e.g., insects) developed new characteristics including larger bodies and the ability to fly. Insects rely mostly on an oxygen diffusion system, with tubes extending from their surface to tracheoles deep within cells. Higher oxygen concentrations would have allowed a greater diffusive penetration and thus, larger bodies.^20^

In addition, the ability to fly was enabled by a hyperoxic atmosphere. The first forms of wings were seen in insects during the Carboniferous (35% oxygen), with wings having mainly a respiratory function and only additionally used for locomotion.^20^ This novel addition to the body form of organisms would have necessitated constantly high oxidative metabolism, which would have been achieved in a hyperoxic environment.^10,20^ Therefore, the addition of wings aided ventilation and minimized the limitations associated with a solely diffusion-based air exchange system. While the exact timing of the evolution of birds, bats and smaller insects is not known, it is estimated that this originated during the mid Jurassic, with some insect gigantism still present at the time. When oxygen levels declined and approached the current 21%, gigantism disappeared and the evolution of novel body forms in flying organisms ended.^10^ Therefore, although large fliers became extinct, improvements in flying organisms allowed their diversification and persisted in different forms in other eras.^20^

Increases and periodical decreases in oxygen concentration have affected evolution and natural selection. Following one of three post-extinction periods, fish and arthropods evolved with an increased ability for oxygen uptake, as they were able to force larger water volumes across their gills, therefore adapting to new oxygen conditions with more effective respiratory systems, thus maximizing their survival.^21^ During the latter Triassic period (220–200 million years ago), mechanisms to maximize respiration efficiency evolved in terrestrial animals, including a novel air-sac system, which evolved in dinosaurs.^21^

The first terrestrial vertebrates are believed to have evolved during a hyperoxic era. With the increasing need for effective gas exchange, gills were not as effective as in aquatic species, with ineffective carbon dioxide discharge, cutaneous respiration was inadequate and more sophisticated lungs for respiration evolved in such vertebrates.^20^

It is estimated that the appearance of the first placental mammals was somewhere between 65 and 100 million years ago, when oxygen levels were high enough to support the Mammalian placental species. High atmospheric oxygen was required to support the transfer of oxygen between maternal arterial blood and placental venous blood.^19^

## Hypoxia effects on whole organisms: Adaptation of species to oxygen changes

The effect of oxygen concentration on organism size and development has been studied in laboratories, using different species. Hyperoxia, defined as above 21% O_2_, can have a positive effect on body size, increasing the size of *Drosophila melanogaster*, whereas hypoxia, O_2_ levels below atmospheric levels (21%), causes the opposite. Furthermore, hypoxia has been shown to decrease the diameter of the trachea and the cell size of these insects.^21,22^

There have been fewer studies on vertebrates, but changes in pO_2_ do affect the evolution of specific traits. In tree frogs exposed to 35% ambient pO_2_, the external gills regressed, whereas early hatching was observed if the animals were returned to ambient oxygen, following exposure to hyperoxia.^23^ In addition, an increase in body weight in hyperoxia-exposed trout was found.^21^

In other experiments, exposure of alligators to 27% oxygen resulted in positive changes on body size, developmental rate and bone composition. Beyond 27% oxygen, however, negative changes were seen, ultimately causing higher mortality rates, indicating a clear role for oxygen in cell signalling and changes in cell phenotype.^21^

Organisms can sense hypoxia, and as a basic response by the action of type I glomus cells in the carotid body, the organism hyperventilates. This limited activation is only in response to environmental hypoxia. It occurs almost immediately, whereas other mechanisms are only initiated if hypoxia is maintained over a period of days. These mechanisms are only effective in cases where oxygen is not limited further than 15%. One of the long-term mechanisms is polycythaemia, which increases the capacity of red blood cells for oxygen, via erythropoietin. Erythropoietin is released by the kidneys and liver and leads to the differentiation and maturation of red blood cell precursors. In humans, its expression is usually minimal and increases in hypoxia, reaching a maximum 48 h after exposure.^24^ There is also an upregulation of 2,3-diphosphoglycerate seen in people who live at high altitude as an adaptation response.^25^

Most mammals and birds require an optimal oxygen tension in order to maintain favourable conditions for cell processes to take place. When these animals are exposed to prolonged hypoxic conditions, the result can be fatal. The brain and heart cannot function due to an ion imbalance of the cell membrane. Under normal conditions, ion equilibrium exists with ions travelling through ATP-dependent channels. For example, the Na/K adenosine triphosphatase (ATPase) channel requires 20%–80% of the cell’s ‘resting metabolic rate’. Cell necrosis under hypoxic conditions occurs as high-energy phosphates decline, membrane depolarisation increases and Ca^2+^ levels increase intra-cellularly causing cell swelling.^26^

Furthermore, animals can be described as oxy-regulators, where their energy consumption rate remains constant irrespective of oxygen availability, or oxy-conformers, where their energy requirements are adjusted depending on oxygen availability.^26^ Tissues and organs can also fall within these groups. For example, the brain is an oxy-regulator and is highly sensitive to hypoxia, therefore unable to adapt to large changes in oxygen, whereas skeletal muscle can adapt to low-oxygen conditions and activate its anaerobic ATP production system, termed the Pasteur effect. Nevertheless, ATP produced under these conditions is not sufficient and can only provide energy for a few minutes in the mammalian brain or a few hours in skeletal muscle.^26^

Other vertebrates such as turtles, fish, amphibians and reptiles can sustain life even under prolonged hypoxia and extreme conditions. It is thought that their ability to survive such long periods of low-oxygen tension is centred on three conditions: (a) the ability to reduce their overall metabolic rate, (b) tolerance of high levels of metabolism by-products, and (c) avoiding or repairing cell injuries following the return to normal oxygen conditions.^27^

Animals that can survive under hypoxia (facultative vertebrate anaerobes) can lower their metabolism by reducing the number of high energy requiring processes and/or by increasing the effectiveness of other processes that ‘produce’ energy. For example, processes such as protein synthesis are inhibited and the energy is allocated to other, more crucial processes such as the Na/K^+^ pump or Ca^2+^ cycling. In general, these animals have the ability to prioritize those functions that are essential and require the most energy during hypoxia.^26^ Therefore, in hypoxia-tolerant animals, ‘channel arrest’ takes place, minimizing the amount of energy required for the maintenance of transmembrane ion gradients. For example, in turtles, there is current flow restriction through Na channels, which then suppresses action potentials, reducing the amount of ATP required.^26,28^

When oxygen supply is adequate, the mitochondria are the site of ATP production. However, if oxygen supply is compromised, the enzyme ATP synthase starts pumping protons from the matrix in order to sustain the membrane potential. This process makes mitochondria highly energy consuming areas, challenging their normal role of energy producers. It is believed that in facultative anaerobes, ATP consumption is limited by inhibiting this enzyme, thereby again conserving energy.^26,28,29^

Other oxygen conserving mechanisms include the higher blood oxygen carrying capacity in some animals, as a result of a higher blood volume and the number of red blood cells.^29^ In addition, a larger body mass in diving mammals could be considered an adaptation to the hypoxic conditions they encounter during deep dives, as their metabolic rate is lower.^29^

During short periods of hypoxia, diving animals have mechanisms in place that help them survive by increasing oxygen storage and supply. Diving animals have a higher concentration of myoglobin in their muscles than other animals, with deep diving animals having the highest levels, shallow divers having intermediate levels and terrestrial animals having the least. This is significant since myoglobin has a higher affinity for oxygen molecules and can act as an oxygen storage facility. Furthermore, myoglobin transports oxygen to the mitochondria and also carries an antioxidant role.^29^

In addition, another important adaptation of divers is quick ventilation. This allows the rapid disposal of the excess CO_2_ and at the same time a quick restock of their oxygen levels. This mainly applies to deep diving mammals. Shallow divers rely more on their oxygen lung capacity and therefore have larger lungs than other species.^29^

Tissue capillary density also varies between animals, with seals for example, having more capillaries in their brains than terrestrial animals. This suggests a mechanism to increase oxygen supply to neural tissue compared to other mammals. Interestingly, in other areas, such as muscle, capillary density seems lower than other terrestrial animals, indicating the importance of the higher myoglobin molecules that are present within them.^29^

At both the whole organism level, tissue level and cell level, therefore, adaptive responses to oxygen aid survival and drive evolution.

## Hypoxia effects on human cells: Oxygen as a regulator of cell function

**Figure 2.**
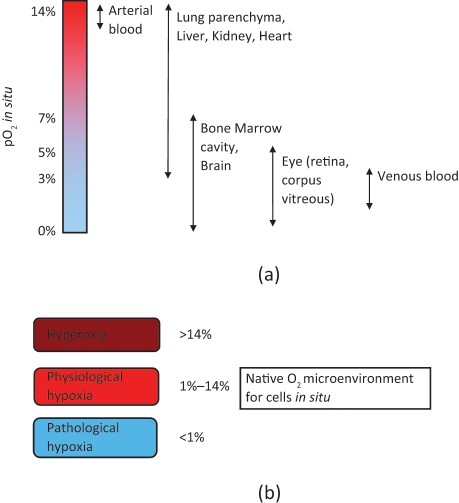
(a) Values of micro-environmental O_2_ found in tissues *in situ* (values taken from Refs. [23,46]). (b) Native O_2_ microenvironment for cells residing within different tissues in the mammalian body.

In adult human tissues, oxygen concentration lies between 1% and 14% pO_2_, depending on the tissue (Figure 2). These values are below the conditions found in air and therefore termed physiological hypoxia. However, levels can fall even further for different reasons such as during embryonic development where tissue growth is greater than the available oxygen supply, or in adulthood during occlusion of arteries. In cases where levels drop below ‘physiological oxygen’ levels, the process of angiogenesis can be initiated.^30^ Of particular importance is the observation that hypoxia is required for the maintenance of stem cell pluripotency.^31^

Hypoxia is a driver of cell function. The effects of hypoxia on cells and tissues are numerous, including embryonic development, maintenance of stem cell pluripotency, induction of differentiation of cell types and the signalling of multiple cascades, including angiogenesis.^32^ Generally, these are regulated by hypoxia-inducible factors (HIFs), prolyl hydroxylases (PHDs), factor-inhibiting HIF-1 (FIH-1), activator protein 1 (AP-1), nuclear factor (NF)-κB, p53 and c-Myc.^33^ There is crosstalk between these transcription factors in response to hypoxia, with the HIFs playing a central role in regulating cellular response to hypoxia.

**Figure 3.**
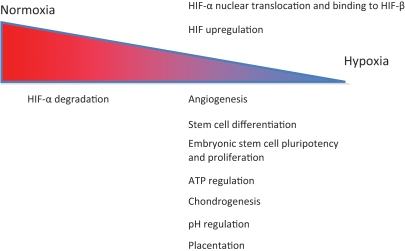
HIF as an oxygen regulator gene. HIF upregulation under hypoxia affects various cell processes. Information from Refs. 32,34–36.

HIF regulates more than 100 genes in all mammalian cells and is also involved in processes such as cell growth and differentiation (Figure 3).^34^ A major factor involved in angiogenesis is vascular endothelial growth factor (VEGF). HIF is a heterodimeric transcription factor that has two subunits, a β subunit, that is not affected by oxygen tension changes and an α subunit, either HIF-1α, HIF-2α or HIF-3α.^35^ HIF-α subunits are usually synthesized and degraded rapidly but are hypoxia inducible, and therefore, under hypoxic conditions their break down is delayed.^30,34,35^ The HIF-α subunits have an ‘oxygen-dependent degradation’ (ODD) domain, whose prolyl residues are hydroxylated in normal oxygen conditions. The hydroxylation then regulates the interaction with the von Hippel–Lindau (VHL) tumour suppressor protein, which leads to HIF-α degradation through ubiquitination. However, under hypoxia the prolyl hydroxylation process is suppressed allowing HIF-α accumulation and nuclear translocation, before binding to HIF-β. The heterodimer can then exert its effects via the hypoxia response elements on its target genes.^34^ HIFs are responsible for the expression of angiogenic molecules in hypoxic tissues that will trigger angiogenesis.^30,36^

HIF-1α and HIF-2α appear to have similar functions, although in some cases they produce different responses to oxygen changes.^35^ A relatively recent discovery suggests that HIF-1α has an important effect on mitochondrial respiration. HIF-1α promotes the expression of pyruvate dehydrogenase kinase, therefore inhibiting the enzymatic activity of pyruvate dehydrogenase.^34^ This prevents the conversion of pyruvate into acetyl CoA, thus preventing pyruvate from entering the Krebs cycle. Therefore, oxygen consumption by the mitochondria is reduced, and reactive oxygen species production is minimized. In addition, a more efficient cytochrome oxidase complex is produced as a result of HIF-1α action, and the ‘normoxic’ version of cytochrome c is degraded by HIF-1α. Thus, HIF-1α action during hypoxia has a role ensuring optimum ATP production and cell integrity, by minimizing reactive oxygen species, during periods of hypoxia.^34^

HIF is generally considered the master oxygen sensor within cells and is central to regulating a cells response to varying oxygen levels. Although the evolutionary origins of HIF are as yet unclear, the recent discovery of HIF in *Trichoplax adhaerens*, the simplest animal, suggests that this early oxygen-sensing mechanism may have evolved during the early Cambrian period.^37^

## Control mechanisms of angiogenesis in oxygen changes

Cells residing within tissues *in situ* require oxygen for basic cell functions. This oxygen level needs to be a fine balance as oxygen is both necessary and yet toxic in large enough volumes. In early development, a vascular system develops, which over the lifespan of an animal, adapts to the changing demands of a tissue (i.e., growth, following muscle hypertrophy or damage to tissues). This process is termed angiogenesis. Angiogenesis *in vivo* involves the activation of endothelial cells, which branch from existing vessels and form new tubes, which will join other capillaries so that they become functional. Furthermore, the extracellular matrix and basement membranes are degraded in order to allow cell migration and expansion through the actions of matrix metallo-proteases (MMPs). Various factors are responsible for controlling the process, with VEGF having one of the most important roles, mainly the initiation of the process.^38^ In addition, other factors such as fibroblast growth factors (FGFs) are involved, having both a direct effect on cells, by promoting mitosis, and indirectly by upregulating VEGF. Platelet-derived growth factor (PDGF) and transforming growth factor β (TGF-β) are involved in recruiting supporting cells to the vessels.^38^ Angiopoietin-1 (ang-1) and angiopoietin-2 (ang-2) are also required, promoting respectively remodelling and maturation of newly formed vessels and endothelial cell sprouting.^38,39^ Finally, angiogenin (ang) promotes endothelial cell and smooth muscle cell migration and proliferation.^40^

Endothelial cells are usually quiescent but have the potential to migrate and divide when exposed to hypoxia or stress; this is when angiogenesis is initiated. Endothelial cells produce the necessary energy for quick cell growth in avascular environments. Although they reside in an environment where oxygen is usually available, these cells rely mostly on anaerobic glycolysis. This provides them with the advantage of being able to survive in conditions of changing oxygen levels. Interestingly, these characteristics make them similar to cancerous cells.^41^ Controlling this process of angiogenesis to provide 3D tissue models with a continuous vascular network throughout them will be critical for the survival of cells deep within 3D tissue constructs, and oxygen is a mediator that can be used to regulate cell behaviour to achieve this.^42^ Oxygen is the main regulator of the process of angiogenesis *in vivo*, via the actions of HIF.^36^ All cells, including endothelial cells are able to sense low oxygen, activating the master oxygen regulator, HIF.^36^ This results in accumulation and nuclear translocation of HIF-α before binding to HIF-β, and this heterodimer can then go on to regulate the angiogenic signalling cascade, which regulates sprouting of vessels and capillaries to supply the tissue with adequate vasculature to increase its oxygen supply.^36,43^

HIFs can affect cell types in different ways, particularly during the process of angiogenesis. It has been suggested that when endothelial cells are exposed to moderate hypoxic conditions, around 5% pO_2_, angiogenic molecules such as vascular endothelial growth factor (VEGF)-A and endothelial nitric oxide synthase (eNOS) are expressed, promoting cell survival, proliferation and migration. On the contrary, if oxygen levels drop further, cell apoptosis can be triggered. Hypoxia has, therefore, two contrasting roles on cell activity, which can be explained by the amount of oxygen provided to these cells.^35^

Furthermore, cell-to-cell junctions and vascular permeability are also affected by hypoxia. The blood–brain barrier is disrupted under hypoxia, and high VEGF-A has been associated with increased vascular leakage, since in studies, blockage with an antibody to VEGF can reduce cerebral oedema.^30,35^

Vascular smooth muscle cells are also affected by hypoxia, with cell apoptosis occurring under extreme hypoxia and cell proliferation under moderate conditions, as well as properties including migration and adhesion. Studies have found a decrease in cell adhesion to the extracellular matrix when HIF-1α expression is increased, which can substantially affect angiogenesis, since the process requires the loosening of these cells from the basement membrane and their neighbouring endothelial cells.^30,35^

## Oxygen and cell differentiation

Oxygen levels greatly influence cell fate. Hypoxia is responsible for determining stem cell fates during embryonic development, during growth and when these cells are recruited in adult life. Bone morphogenesis is one of the processes affected by oxygen concentration in the surrounding environment. In the epiphyseal growth plate of a developing long bone, the oxygen tension within the pre-hypertrophic region (region furthest away from vascular supply) is 21 mmHg (2.7%) compared to 57 mmHg (7.5%) within the hypertrophic region, which is where a good vascular network is found.^44^ The cells in the pre-hypertrophic region remain more proliferative and more ‘stem cell like’, compared to the hypertrophic region, where oxygen levels are higher and cells become terminally differentiated.^45^

Human bone marrow stem cells (HBMSCs) reside within an area of low oxygen; with suggestions ranging from 4%–7% to 1%–2%, substantially lower than the oxygen level of surrounding tissues.^31,46^ This low-oxygen state is now believed to be a mechanism by which stem cells maintain pluripotency. It has been proposed that the ability of stem cells to divide (self-renew) is dependent upon the hypoxic environment. For example, in low-oxygen environment, where there is a limited energy supply, there is strict replication of DNA without the activation of multiple transcriptional differentiation programs.^31^ As well as this, HBMSCs are able to use anaerobic glycolysis for their energy needs, making them an ideal candidate for transplantation into ischaemic tissues. Furthermore, HIF targets glucose-6-phosphate transporter, which controls gluconeogenesis, increasing the availability of glucose to the cells and hence, increasing their survival under hypoxia. Cell death will occur if hypoxia is extended, mainly due to mitochondrial dysfunction, lactate build-up and pH changes.^46^

Due to the ability of HBMSCs to differentiate into osteoblasts, chondrocytes, myocytes and adipocytes, various research have examined the effect of oxygen on their proliferation and differentiation potential under varying oxygen conditions.^46^ A body of evidence suggests that HBMSC proliferation is increased when cells are cultured under low-oxygen conditions, which more closely resembles their original environment rather than atmospheric conditions of 21%.^46^

Cell differentiation is also affected by oxygen changes with some contrasting evidence (reviewed in Ref. 46). Most commonly, osteogenesis is assessed where there is evidence of mineralisation by differentiated HBMSCs cultured at around 5% for all or part of the culture.^47,48^ There are studies contrary to this, where a decrease in mineralisation has been observed when culturing cells in hypoxia.^49,50^ These differences may be attributed to our limited assessment of when cultures have actually differentiated, and general differences in the way we culture our cells.

Chondrogenic differentiation is enhanced when BMSCs are cultured from between 1% and 5% pO_2_, assessed by increases in sex-determining region Y (SRY)-box 9 (SOX9) and other targets via HIF-1α, increasing glycosaminoglycan and collagen type II production.^51,52^ Adipogenic differentiation is inhibited if cells are exposed to 1%–2% pO_2_. Cells cultured in such conditions will remain in an undifferentiated state and proliferate until returned to normoxia. This implies a role of hypoxia in maintaining cells in a proliferative state, instead of promoting cell differentiation.^32^

## Oxygen and embryonic development

A high percentage of toti- and pluri-potent stem cells within a developing embryo are also maintained in lower oxygen tensions, which is coherent with arguments relating to BMSCs. In the first trimester of pregnancy, it is estimated that human placentas function in around 2.4% oxygen pressure, in contrast to the neighbouring endometrium, where levels are around 5.3%. Therefore, the embryo develops under hypoxic conditions, around 2%.^32^ Embryonic stem cells exposed to hypoxia show more efficient growth, and culturing these cells at 3%–5% oxygen results in fewer differentiated cells than cells exposed to ambient oxygen.^45^

There is a body of evidence suggesting that hypoxia is a necessary prerequisite for the maintenance of embryonic stem cell pluripotency.^32,45^ Embryonic stem cells exposed to hypoxia show more efficient growth, with experiments using bovine blastocysts grown in a low-oxygen environment showing an increase in the number of inner mass cells compared to animals grown in higher oxygen concentrations.^45^ There is evidence therefore for both embryonic and adult stem cells of a role for hypoxia in maintenance of pluripotency.

Studies in mice have shown the importance of HIF during embryonic development. Deletions of HIF-1α or HIF-1β cause the death of the animals around day 10 of gestation. HIF-1α mutations cause death due to cardiovascular problems, neural tube defects^45^ and mesenchymal stem cell loss.^32^ HIF-1β absence is also lethal, with similar problems, in addition to somite defects, branchial arches and placental defects.^45^

Oxygen also appears to affect placentation in mammals. In mouse embryos, glycolysis is the ATP supplying pathway until day 9.5 of gestation. Following day 10.5, however, the placenta is formed, which is thereafter responsible for supplying the growing foetus with oxygen and nutrients. Deletions of HIF-1α and HIF-2α in mice result in an abnormal placenta, with fewer blood vessels and abnormal histological characteristics.^45^ In human pregnancies, the placenta forms early and is then exposed to changes in oxygen concentration. However, it is able to adapt to these changes and provides the foetus with the necessary nutrients at the same time as it protects it from oxidative stress.^53^

Furthermore, oxygen concentrations also affect proliferation, invasion and differentiation of cytotrophoblast cells as they invade the uterine spiral arterioles.^32^ Evidence suggests that a gradient of oxygen, from approximately 2.5% pO_2_ to 5% pO_2_, guides the process. Low pO_2_ is found at the site proximal to the placenta, which allows proliferation of the cells, but as these enter the site of high pO_2_ close to the spiral arterioles, differentiation to an invasive phenotype begins, allowing their anchoring to the endometrium.^32^

Another tissue that is greatly influenced by oxygen concentrations in development is cartilage. Cartilage is a hypoxic tissue, due to its avascular nature. Mesenchymal stem cells form condensations during embryonic development within the area of cartilage formation and are exposed to hypoxic conditions whilst they differentiate into the cartilaginous phenotype. Studies have shown the importance of maintaining low-oxygen conditions during this process, through knockout experiments of HIF in mice. HIF-1α deletions later in development result in chondrocyte apoptosis, whereas earlier in development embryos develop abnormal joints. Therefore, HIF and its downstream genes have important roles during joint formation.^32^

## Culturing cells in 3D scaffolds for tissue engineering

Traditional cell culture techniques involve growing cells optimally in a systematic, controlled manner, easily reproduced in labs across the world. This has meant cells are grown on 2D tissue culture plastic and at atmospheric oxygen (these oxygen levels are higher than the *in vivo* setting, which to a cell translates to hyperoxia or oxidative stress). Both these parameters are not the native microenvironment of cells in tissues. To accurately depict cell behaviour and responses to stimuli, these basic parameters should ideally be recapitulated. Further complexities, in terms of the importance of multiple cell types, appropriate extracellular matrix composition, defined nutrient availability, etc., can then be added to systematically introduce tissue complexity when studying cell behaviour.

In the field of tissue engineering, recapitulating the 3D architecture, the complex cell–cell interactions, as well as the introduction of vascular and neural networks within tissues is of paramount importance, if the ‘tissue’ produced is to be of any benefit. For this, a much more physiological approach has been taken within the tissue engineering field, where the minimum parameters required are culturing in 3D, use of native matrix proteins, and perfusion bioreactors, which continuously circulate media to keep cells supplied with appropriate levels of oxygen and other nutrients.^54,55^ There is also a current emerging view that culturing cells in physiological hypoxia will predictably influence certain cell responses, for example, in upregulating angiogenic protein cascades and influencing stem cell differentiation.^31,42,56^

**Figure 4.**
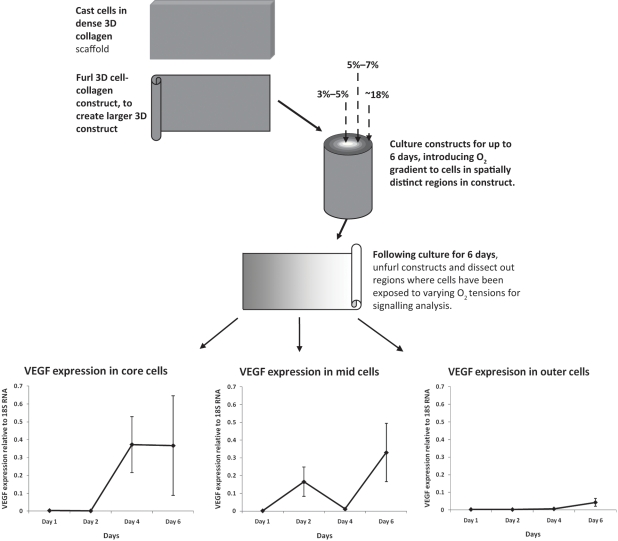
Culturing cells in 3D constructs, with mechanisms to introduce O_2_ gradients and methods to test specific populations of cells exposed to these varying O_2_ tensions. Specific VEGF signalling by cells in different positions exposed to varying O_2_ tensions. Angiogenic signalling graphs taken from Ref. 60.

By culturing cells in 3D to build tissue models, it is possible to test cell behaviour in a physiologically relevant set-up.^55,57^ In particular, the culture of cells within 3D scaffolds allows for natural oxygen consumption gradients to form from the surface to the core (high to low oxygen) of 3D constructs.^58,59^ These gradients are dependent upon the cell type and density. By utilizing 3D culture systems, it is possible to decipher an understanding of oxygen-specific cell signalling, dependent upon the spatial position of cells, that is, core cells versus surface cells. For this, cells are cultured in 3D constructs, and following monitoring of oxygen in precise locations, these cell populations exposed to varying oxygen tensions can be dissected and their signalling studied (Figure 4).^60^ Such 3D culturing techniques offer insight into the optimal oxygen levels under which to culture cells to drive certain regimens of signalling. For example, by culturing BMSCs in 3D collagen scaffolds and monitoring, in real- time, the oxygen levels from the surface to the core, we can see that when cultured at between 3% and 5% oxygen, these cells optimally upregulate VEGF, compared to 18%, where VEGF is not significantly upregulated (Figure 4).^60^

By relying upon the natural trigger of angiogenesis, oxygen, to control cell signalling cascades, we can controllably observe and study this angiogenic factor cascade to gain further insights into this signalling, as well as utilize this system to control endothelial cell behaviour to form vascular networks *in vitro*.^56^

These same 3D cultures can be used to study a multitude of cell responses to varying oxygen, in particular the influence of oxygen tension on stem cell phenotype. The formation of such oxygen gradients occurs within all 3D tissue-engineered scaffolds and provide uniquely controlled systems in which to study specific cell behaviours.^61^

## Implications for cell therapies

There is currently a huge interest and surge in the use of cell-based therapies for the repair or regeneration of tissue affected by ageing or trauma. Therapies involving direct injection of cells into ischaemic or damaged tissues for enhancing repair are also subject to the effects of lowered oxygen level. When large numbers of cells are transported in small volume syringe barrels, the implications for cells are transport and deposition within a severely restricted oxygen environment.^62,63^ Although oxygen levels have not been measured in such scenarios, one would expect these levels to drop below normal physiological hypoxia. As well as this, following direct injection into a tissue (current therapies include injection into bone, tendon, cartilage and heart to name a few), these cells take time to integrate with tissues and may not have a direct contact with a vascular supply; indeed, their fate and localisation is a subject of debate, and techniques to track this cells *in vivo* are currently under investigation. In the meantime, it is unclear what percentage of cells survives in such scenarios, and whether reported angiogenesis to such tissues is actually due to cell presence or hypoxia-generated cell signalling of angiogenic factors being released. Understanding the effects of hypoxia on cell signalling will lead to a better understanding of the effects of these cells when injected *in vivo* for clinical applications.

## Conclusion

In this review, we have discussed oxygen in different contexts: oxygen and evolution, species adaptation to oxygen changes, the effect of hypoxia on cells and cell processes. Oxygen is crucial for cell survival and changes in its concentration influence cell fates, cell proliferation, pH, angiogenesis and other processes mainly through HIF and its downstream factors. Oxygen is not merely an element important for survival for its role in energy production, but it has a much more complex role in regulating life. Its ‘power’ to influence organisms can be deduced from both its role in evolution, species adaptation mechanisms to changing oxygen concentrations and to the current focus on its effects on different cells and cell processes.

With specific reference to tissue engineering and regenerative medicine, oxygen plays a critical role in the proliferation and differentiation of cells, particularly cells of ‘stem cell’ origin, which is a tool that can be utilized to control the formation of specific tissues from a uniform cell source. By developing more sophisticated tissue models, we will be able to decipher more accurately the roles of oxygen in determining cell signalling and cell phenotype. The angiogenic cascade is an example of a process that can be controlled using oxygen as an environmental stimulus, to enhance vascularisation of constructs both *in vitro* and *in vivo*. For engineering complex tissues *in vitro*, we can utilize the same oxygen response system that controls specific cell behaviours *in vivo*, in a more physiologically complete manner. Using native micro-environmental cues to control cell behaviour is likely to yield physiologically relevant responses.
